# Association between Consultation by a Comprehensive Integrated Palliative Care Program and Quality of End-of-Life Care in Patients with Advanced Cancer in Edmonton, Canada

**DOI:** 10.3390/curroncol30010068

**Published:** 2023-01-09

**Authors:** Cara Robertson, Sharon M. Watanabe, Aynharan Sinnarajah, Alexei Potapov, Viane Faily, Yoko Tarumi, Vickie E. Baracos

**Affiliations:** 1 Division of Palliative Care Medicine, Department of Oncology, University of Alberta, Edmonton, AB T6G 1Z2, Canada; 2 Division of Palliative Medicine, Queen’s University, Kingston, ON L1G 2B9, Canada; 3 Medical College of Wisconsin Affiliated Hospitals, Milwaukee, WI 53226, USA

**Keywords:** palliative care, end of life care, quality indicators, aggressive care

## Abstract

Literature assessing the impact of palliative care (PC) consultation on aggressive care at the end of life (EOL) within a comprehensive integrated PC program is limited. We retrospectively reviewed patients with advanced cancer who received oncological care at a Canadian tertiary center, died between April 2013 and March 2014, and had access to PC consultation in all healthcare settings. Administrative databases were linked, and medical records reviewed. Composite score for aggressive EOL care was calculated, assigning a point for each of the following: ≥2 emergency room visits, ≥2 hospitalizations, hospitalization >14 days, ICU admission, and chemotherapy administration in the last 30 days of life, and hospital death. Multivariable logistic regression was adjusted for age, sex, income, cancer type and PC consultation for ≥1 aggressive EOL care indicator. Of 1414 eligible patients, 1111 (78.6%) received PC consultation. In multivariable analysis, PC consultation was independently associated with lower odds of ≥1 aggressive EOL care indicator (OR 0.49, 95% CI 0.38–0.65, *p* < 0.001). PC consultation >3 versus ≤3 months before death had a greater effect on lower aggressive EOL care (mean composite score 0.59 versus 0.88, *p* < 0.001). We add evidence that PC consultation is associated with less aggressive care at the EOL for patients with advanced cancer.

## 1. Introduction

Recent developments in oncological therapies have offered more options to patients with advanced cancer [1,2]. However, evidence shows that patients are increasingly exposed to aggressive care at the end of life (EOL), leading to poorer outcomes, including decreased quality of life, decreased care satisfaction, more complex bereavement for survivors and increased health care utilization and costs at the EOL [2,3,4,5,6]. Earle et al. and others have developed quality indicators based on administrative data to aid with identifying aggressive care at the EOL. These indicators have been adopted as markers for potentially inappropriate care, increasing patient burden and cost of care, with little to no benefit [7]. They are intended for use at a health systems level, as they are unable to assess care in individual circumstances. Many markers of aggressiveness of care have been proposed [7,8] and several have been validated [2,8]. The American Society of Clinical Oncology Quality Oncology Practice Initiative and National Quality Forum have endorsed or utilized such measures for surveillance or quality improvement purposes. These include ≥2 emergency room (ER) visits, ≥2 hospital admissions, hospitalization duration >14 days, and ICU admission in the last 30 days of life, chemotherapy administration in the last 14 days of life, and death in hospital [2].

Palliative care provided concurrently with cancer care has been shown to improve multiple clinical outcomes for patients with advanced illness, including symptom burden, quality of life, care satisfaction, and survival [9,10,11]. Specialist palliative care has been shown to have a greater effect size if implemented early in the advanced cancer trajectory [12,13]. The association between palliative care and aggressive care at the EOL has not been studied as extensively [4,10]. Abedini et al. conducted a systematic review that focused on interventions to reduce aggressive care at the EOL, which found that studies of palliative care specialty referral did not show consistent reductions and had methodological limitations. Meta-analysis was not possible due to high heterogeneity between studies [8].

The Cross Cancer Institute (CCI) is the only tertiary cancer center in the northern half of the province of Alberta in Canada. It is located in the city of Edmonton. Its comprehensive care includes oncological consultation, systemic and radiation treatments, education, and research in both inpatient and outpatient settings. Specialist palliative care consultation is available for both inpatients and outpatients on site at the CCI. Referral criteria include uncontrolled symptoms and/or need for discharge to community-based palliative care due to lack of treatment options. During the study period, referral was at the discretion of treating oncologists. We have previously published on the frequency, timing and predictors of palliative care consultation at the CCI [14].

In addition to CCI-based consultation, patients who reside in the Edmonton Zone (i.e., health region) have access to a well-established comprehensive integrated palliative care program. It is comprehensive in its coverage of the full spectrum of care settings (cancer center, acute care, community), and integrated in the sense that it is centrally coordinated, with application of common standards and outcome evaluation [15]. Referrals are received from primary care or specialist providers for consultation on symptom control, coordination of community-based palliative care resources or admission to inpatient palliative care settings [16]. Consultation is performed by palliative care specialist physicians, nurses, and pharmacists, with participation of dieticians, occupational therapists, physiotherapists, psychologists, social workers, spiritual care professionals and other disciplines as required. Assessment tools used throughout the program include the Edmonton Symptom Assessment Scale-Revised [17], Edmonton Classification System for Cancer Pain [18] and Palliative Performance Scale [19]. The palliative care program at the CCI is a component of this greater program and its consultation procedures are aligned. This integrated organization of palliative care services differs from most of the settings in which aggressive care at the EOL has been studied, which have largely been limited to within-cancer center [10], within-hospital [20] or home-based care in the community [21].

Therefore, considering the heterogeneity of the evidence, as well as limited studies in the context of a comprehensive integrated specialist palliative care program in Canada, we undertook a retrospective study with the aim of exploring the association between occurrence and timing of palliative care consultation with aggressive care at the EOL in patients with advanced cancer. Our hypothesis was that patients who receive palliative care consultation, especially earlier in the disease trajectory, are less likely to receive aggressive care at the EOL.

## 2. Materials and Methods

We conducted secondary analysis of routinely collected health data, linking data from the Alberta Cancer Registry, electronic medical records, Edmonton Zone Palliative Care Program database, Ambulatory Care Database and Hospital Discharge Abstract Database. Approval to conduct the study was granted by the Health Research Ethics Board of Alberta—Cancer Committee.

### 2.1. Patient Inclusion and Exclusion Criteria

**Figure 1:**
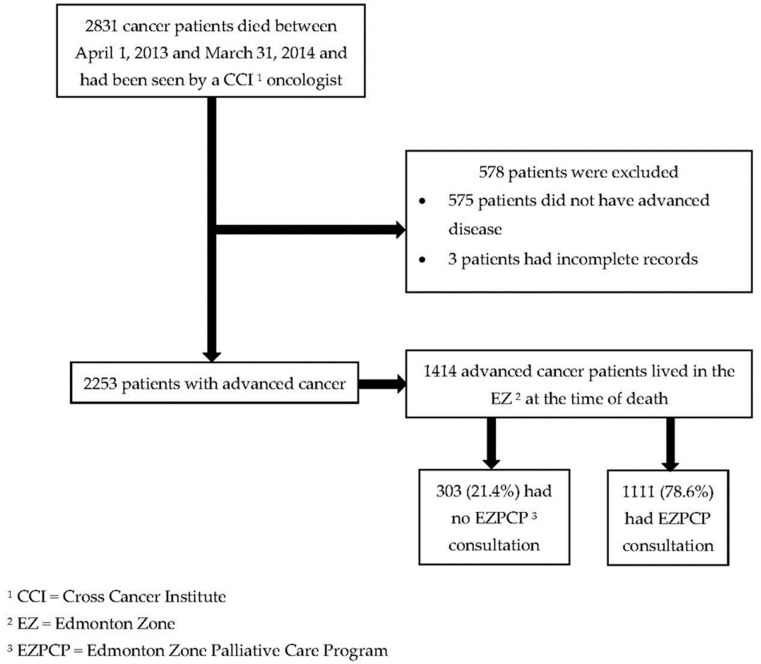
Patient flow chart.

Patients were drawn from a population compiled for our previously published study of frequency, timing and predictors of palliative care consultation at the CCI [14]. Eligible patients were those who had seen an oncologist at the CCI for advanced cancer, died between 1 April 2013 and 31 March 2014, lived in the Edmonton Zone at the time of death, and were ≥18 years of age. Patients were excluded if they did not advanced disease while under oncological care, had incomplete records, and did not live in the Edmonton Zone at the time of death (Figure 1). Patients residing in other health care zones were not included as the palliative care programs in these predominantly rural areas were organized differently and we did not have access to program data.

### 2.2. Data Sources

Firstly, the Alberta Cancer Registry was used to identify patients with cancer who had died during the study period. Date of initial oncological consultation, age, sex, marital status, cancer type, chemotherapy usage, healthcare zone of residence and postal codes were extracted. Postal codes of patients’ last known living location were used to determine income level, as per Statistics Canada [22].

Secondly, date of diagnosis of advanced cancer was determined by electronic medical record review. Advanced cancer was defined as incurable disease or potentially curable disease but patient unable or unwilling to receive curative treatment. Incurability was defined according to criteria outlined in Alberta Cancer Guidelines for treatment of different cancer types [23]. In records where there was uncertainty about the diagnosis of advanced cancer, oncologists’ consultation and progress notes in CCI electronic medical records (ARIA MO^®^) were examined for mention of incurability.

Thirdly, occurrence and date of consultation with a palliative care team (at the CCI or other Zone location) was collected from the Edmonton Zone Palliative Care Program electronic database. 

Information on hospital visits, including ER visits, hospital admission, ICU admission and death in hospital was collected from the Ambulatory Care Database and Hospital Discharge Abstract Database. Frequency of indicators of aggressive care at the EOL was determined.

### 2.3. Statistical Analysis

The study population was characterized using descriptive statistics, comparing patients who did and did not receive palliative care consultation. We used for *t*-test for continuous variables and Pearson’s χ^2^ test for categorical variables. Normal distribution of average and applicability of *t*-test is ensured by sufficient length of the data sets [24].

A composite score (score range 0–6) for aggressive care at the EOL was determined as per Hui et al. [10] by assigning one point each for the following: ≥2 ER visits, ≥2 hospital admissions, hospitalization duration >14 days, ICU admission, and chemotherapy administration (any systemic antineoplastic agent at any cycle) in the last 30 days of life, and death in hospital.

A multivariable logistic regression analysis was performed with the dependent variable of ≥1 aggressive EOL care indicator and independent variables of age, sex, income, cancer type, interval from advanced cancer diagnosis to death, and palliative care consultation. 

Statistical analysis was done in R [25], binomial logistic regression was implemented using glm function. A *p*-value of ≤0.05 was considered significant.

## 3. Results

Of 1414 eligible patients with advanced cancer who received oncological care at the CCI and lived in the Edmonton Zone, 1111 (78.6%) received palliative care consultation (Figure 1). Patients who received palliative care consultation were younger (69.1 years versus 71.5 years, *p* = 0.003) and had a different distribution of cancer types than those who did not (Table 1). Sex, marital status and income were not significantly different between the two patient groups. For patients who received palliative care consultation, the first site of consultation was the CCI in 45.6% of cases. Patients received palliative care consultation a median of two months (interquartile range 1–5 months) prior to death (Table 1).

**Figure 2:**
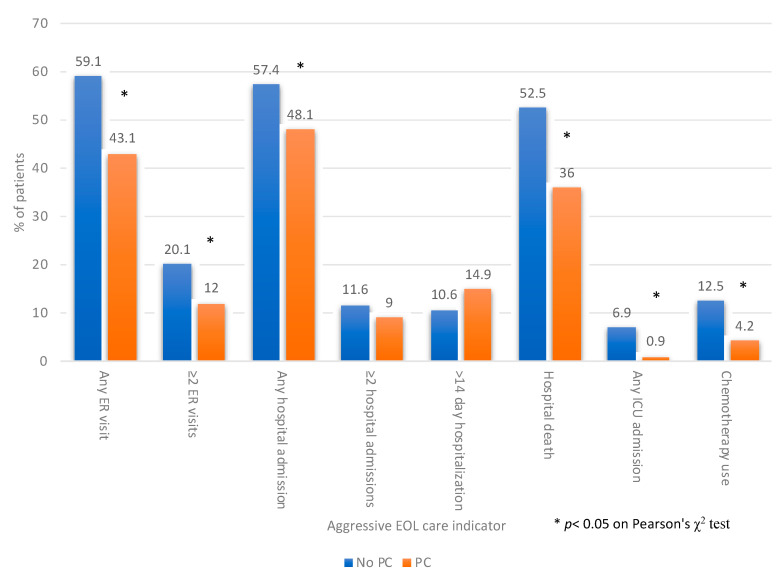
Proportions of indicators of aggressive care at the end of life (EOL) in last 30 days of life, based on occurrence of palliative care (PC) consultation (*N* = 1414).

**Figure 3:**
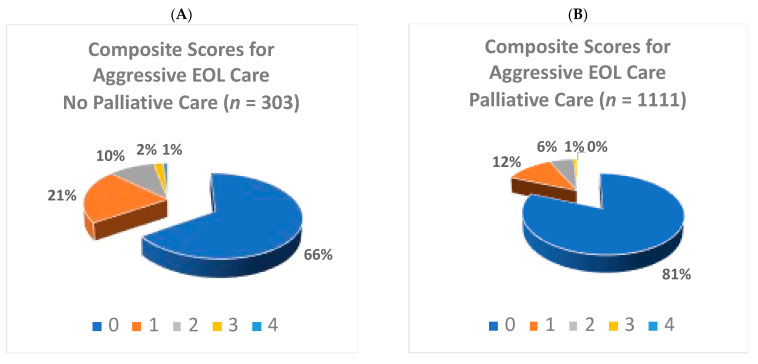
Proportions of composite scores for aggressive care at the end of life (EOL) (scale 0–6) [10], based on occurrence of palliative care consultation (Panel **A** = No; Panel **B** = Yes) (*N* = 1414).

The proportions of patients with indicators of aggressive care at the EOL were lower for those who received palliative care consultation than those who did not (Figure 2). Patients who received palliative care consultation were less likely to visit an ER (43.1% versus 59.1%, *p* < 0.001) or have ≥2 ER visits (12.0% versus 20.1%, *p* < 0.001). They were less likely to be admitted to hospital (48.1% versus 57.4%, *p* = 0.005), have a hospital death (36% versus 52.5%, *p* < 0.001) or be admitted to ICU (0.9% versus 6.9%, *p* < 0.001). They were less likely to have chemotherapy in the last 30 days of life (4.2% versus 12.5%, *p* < 0.001) or during the last 14 days of life (1.2% versus 6.3%, *p* < 0.001). Occurrence of ≥2 hospital admissions (9% versus 11.6%, *p* = 0.22) or a prolonged (≥14 days) hospital stay (14.9% versus 10.6%, *p* = 0.7) was not significantly different between patients who did and did not receive palliative care consultation. Mean composite aggressive EOL care score was lower in patients who received palliative care consultation compared to those who did not (0.26 versus 0.51, *p* < 0.001). The distribution of composite scores is depicted in Figure 3.

**Figure 4:**
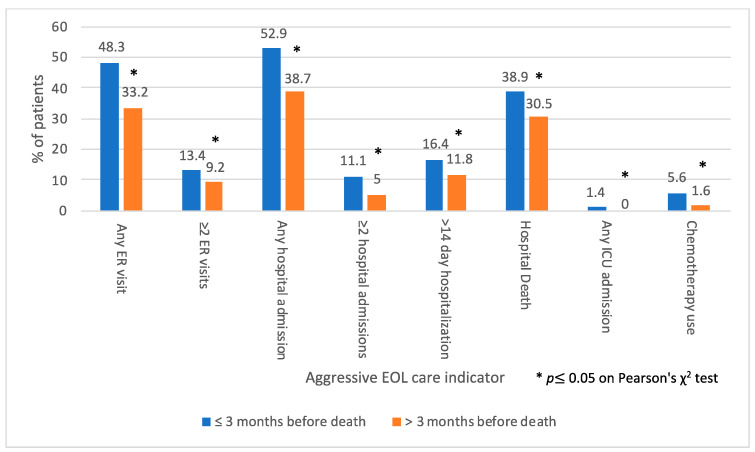
Proportions of indicators of aggressive care at the end of life (EOL) in last 30 days of life, based on timing of palliative care (PC) consultation (*N* = 1414).

Receiving palliative care consultation >3 months before death compared to receiving it ≤3 months before death was associated with lower proportions of patients with indicators of aggressive care at the EOL (Figure 4). These patients were less likely to visit an ER once (33.2% versus 48.3%, *p* < 0.001) or two or more times (9.2% versus 13.4%, *p* = 0.05). They had a lower frequency of any hospital admission (38.7% versus 52.9%, *p* < 0.001), two or more hospital admissions (5% versus 11.1%, *p* = 0.001), prolonged (≥14 days) hospital stay (11.8% versus 16.4%, *p* = 0.05), ICU admission (1.4% versus 0%, *p* = 0.05) or chemotherapy use in the last 30 days of life (1.6% versus 5.6%, *p* = 0.003) and hospital death (30.5% versus 38.9%, *p* = 0.02). The mean composite score for aggressive EOL care in the patient group receiving early (>3 months before death) palliative care consultation was lower than in that receiving later palliative care consultation (≤3 months before death) (0.15 versus 0.31, *p* < 0.001).

Multivariable logistic regression analysis for the presence of at least one indicator of aggressive care at the EOL (Table 2) showed that palliative care consultation (OR 0.49, 95% CI 0.38–0.65, *p* < 0.001), older age (OR 0.97, 95% CI 0.97–0.98, *p* < 0.001), and female sex (OR 0.61, 95% CI 0.48–0.79, *p* < 0.001) were associated with lower likelihood of receiving any aggressive care at the EOL, whereas hematological malignancy was associated with higher likelihood of receiving aggressive care at the EOL (OR 1.34, 95% CI 1.06–2.69, *p* = 0.02).

## 4. Discussion

In this study, we evaluated the association between occurrence and timing of specialist palliative care consultation and indicators of aggressive care at the EOL, in patients with advanced cancer who received care at a tertiary cancer center and had access to a comprehensive integrated palliative care program. We demonstrated that the occurrence of a palliative care consultation was associated with lower proportions of patients with indicators of aggressive care at the EOL. Furthermore, we demonstrated that earlier palliative care consultation was associated with potentially better quality of EOL care compared to later consultation, with lower proportions of patients with ER visits, hospital admissions, ICU admissions, prolonged hospital stays, and chemotherapy usage in the last 30 days of life, and death in hospital. Our setting maximizes accessibility to specialist palliative care consultation, within and beyond the tertiary cancer center, with 78% receiving consultation overall.

The review of Abedini et al. in 2019 pointed to several trials suggesting that specialist palliative care was associated with decreased aggressiveness of care at the EOL but lacked overall consistency in that conclusion. The authors also noted high heterogeneity of study design, precluding meta-analysis. Subsequent to this review, several studies favoring palliative care’s association with decreased aggressiveness of care at the EOL have emerged [20,21,26,27]. For example, Conlon et al. conducted a propensity matched cohort study using administrative data of an ambulatory service at a cancer center in Sudbury, Canada and concluded that the palliative care group had lower rates of nearly all indicators of aggressive care at the EOL (absolute risk reduction 12.73%, 95% CI 12.65–12.81). The largest difference was observed for dying in hospital (absolute risk reduction 19.89%, 95% CI 19.78–20.00, relative risk 0.55, 95% CI 0.47–0.64) [26].

Our study adds evidence that any specialist palliative care consultation (even if done late in disease trajectory) is associated with less aggressive care at the EOL. 34.3% of patients who did not receive palliative care consultation had at least 1 indicator of aggressive care at the EOL (i.e., composite score of ≥1) compared to 18.6% patients who received palliative care consultation. Thus, the majority of patients in our context did not receive aggressive care at the EOL, even with standard oncological care. This compares favorably with studies based in the United States, where up to half of patients received aggressive care at the EOL and may be reflective of regional variation and the difference in health care system models [10,28].

The concept of earlier referral to palliative care conferring additional benefit is becoming well established [9,10,11,12,13,29]. However, the concept of “early” or “timely” remains poorly defined, and studies looking at this have a high degree of heterogeneity. In our study, we defined early palliative care as more than three months before death. Earlier consultation was associated with a lower composite score for aggressive EOL care compared to later consultation. This is similar to Hui et al.’s findings in 2014 in a cancer center in Texas but different from de Oliveira Valentino et al.’s findings in a Brazilian cancer hospital [10,30]. During the period of our study, referral to palliative care was done at the discretion of the treating oncologist. There is no automatic referral pathway for patients who have a diagnosis of advanced cancer in our palliative care program. In a landmark study, Temel et al. randomized patients to receive palliative care consultation within eight weeks of diagnosis of advanced lung cancer versus standard care. The intervention group survived significantly longer, had better quality of life, and received fewer aggressive treatments at the EOL. This study has led to discussion as to whether palliative care consultation should be considered standard of care in advanced cancer [11]. It is also important to consider that data collection for our study occurred between 2013 and 2014, not long after the Temel study. As such, referral patterns may not yet have changed accordingly. Unfortunately, more recent data was not available to us. ASCO published a guideline in 2016 that recommends that all patients diagnosed with advanced cancer be referred for palliative care consultation within eight weeks of diagnosis of advanced disease, based on the above-mentioned study, as well as several other randomized control trials [9]. Integration of palliative care into standard oncologic care is associated with a lower frequency of indicators of aggressive care at the EOL [10,13,28]. Our study provides further evidence of this.

There are several possible explanations why earlier referral to palliative care and integration of palliative care into oncologic care is associated with improved quality of care at the EOL. Firstly, the opportunity to build a longitudinal relationship with the palliative care team is given more time, allowing development of more trust and improved communication. This in turn, allows for effective advance care planning discussions, thereby reducing decisions to pursue aggressive care at the EOL. Secondly, earlier detection and active management of symptoms decreases the likelihood of symptom crises necessitating ER visits and hospital admissions. Thirdly, involvement of palliative care can increase access to community-based resources, such a palliative home care and inpatient hospice, decreasing the need for hospital admission and associated aggressive treatments [10,11].

Another finding is that females were less likely to receive any aggressive care at the EOL, which is consistent with other studies [10,31,32]. A prospective study of patients with advanced cancer demonstrated a higher rate of ICU care at the EOL in males compared to females, but no sex differences in the occurrence of EOL discussions. Possible explanations for the results include sex differences in the content of EOL care discussions, how patients use the information in EOL care decision-making, and physician recommendations on EOL care or patient perceptions of them [32].

Our study has a number of strengths. Secondary analysis of routinely collected data minimizes recruitment bias. It is one of a few studies looking at the association of provision and timing of palliative care consultation on indicators of aggressive care at the end of life in the Canadian context, particularly in the setting of a comprehensive integrated palliative care program. The study is unique in that it included patients who were confirmed to have advanced cancer by review of medical records (i.e., who would have been eligible for referral for palliative care consultation), rather than patients who died with cancer which may or may not have been at an advanced stage. The study has a large sample size, and the data set is complete. There are several limitations to this study. The retrospective nature of this research means that causality cannot be inferred. Furthermore, indicators of aggressive care look at interventions with limited clinical utility in general, but it is not possible to know whether the interventions were clinically indicated in specific situations or congruent with individual patients’ needs and values. Patients’ wishes regarding resuscitative care could be a confounding factor, as patients who desire such care may be less likely to be referred to palliative care and more likely to receive aggressive care at the EOL; unfortunately, documentation of goals of care was paper-based during the study period and not retrievable. The data were collected at a single, urban tertiary center and may not reflect regional variation accurately.

## 5. Conclusions

This study adds evidence that palliative care consultation, especially earlier in the advanced cancer trajectory, is associated with less aggressive care at the EOL. It lays a foundation for future prospective research to assess the effect size, influence of potential confounders such as goals of care, and clinical implications in the care of patients with advanced cancer. Findings from this study informed the Palliative Care Early and Systematic (PaCES) project, an Alberta-based implementation study examining systematic provision of early palliative care for patients living with advanced cancer [33].

## Figures and Tables

**Table 1:** Patient characteristics based on any EZPCP ^1^ consultation (*N* = 1414).

Patient Characteristics	No Palliative Care Consultation (*n* = 303)	Palliative Care Consultation (*n* = 1111)	*p*-Value ^2^
Age in years, mean	71.5	69.1	**<0.01**
Female sex (%)	139 (45.9)	539 (48.5)	0.45
Married (%)	178 (58.7)	669 (60.2)	0.69
Income (%)			
Lowest Quintile	68 (22.4)	243 (21.9)	0.98
Highest Quintile	53 (17.5)	199 (17.9)	
Cancer type (%)			
Breast	17 (5.6)	100 (9)	**<0.01**
Gastrointestinal	40 (13.2)	301 (27.1)	
Genitourinary	29 (9.6)	116 (10.4)	
Gynecologic	14 (4.6)	81 (7.3)	
Head & Neck	12 (4)	38 (3.4)	
Hematologic	42 (13.9)	64 (5.8)	
Nervous System	11 (3.6)	34 (3.1)	
Other	21 (6.9)	61 (5.5)	
Respiratory	117 (38.6)	316 (28.4)	
Site of first palliative care consultation (%)	N/A ^3^		
Tertiary cancer center		507 (45.6)	
Tertiary hospital		314 (28.3)	
Community (including community hospital)		290 (26.1)	
Months between advanced cancer diagnosis and death, median (interquartile range)	6 (2–17.5)	9 (4–21) ^4^	0.11
Months between advanced cancer diagnosis and first palliative care consultation, median (interquartile range)	N/A	4 (1–15)	N/A
Months between first palliative care consultation and death, median (interquartile range)	N/A	2 (1–5)	N/A

^1^ EZPCP = Edmonton Zone Palliative Care Program; N/A = Not Applicable. ^2^ *p*-value determined using *t*-test for continuous variables and Pearson’s χ^2^ test for categorical variables; statistically significant values in bold. ^3^ N/A = Not applicable. ^4^ (Q1–Q3) interval.

**Table 2:** Multivariable logistic regression analysis for presence of ≥1 indicator of aggressive care at the EOL ^1.^(*N* = 1414).

Effect	OR ^2^	Lower CL ^3^	Upper CL	*p*-Value ^4^
Age, per year	0.97	0.97	0.98	**<0.01**
Female sex	0.61	0.48	0.79	**<0.01**
Breast cancer	0.92	0.59	1.45	0.72
Gastrointestinal cancer	0.82	0.61	1.10	0.18
Genitourinary cancer	0.75	0.50	1.12	0.16
Gynecological cancer	0.91	0.56	1.47	0.68
Head and neck cancer	0.64	0.35	1.18	0.15
Hematological cancer	1.34	1.06	2.69	**0.02**
Nervous system cancer	0.59	0.03	1.13	0.11
Other cancer	0.83	0.51	1.36	0.46
Income quintile 2	0.93	0.67	1.29	0.65
Income quintile 3	1.20	0.86	1.67	0.27
Income quintile 4	0.84	0.60	1.20	0.34
Income quintile 5	0.83	0.59	1.17	0.29
Advanced cancer to death in months	1.00	0.99	1.00	0.18
EZPCP ^5^ consultation	0.49	0.38	0.65	**<0.01**

^1^ EOL = End-of-Life. ^2^ OR = Odds ratio. ^3^ CL = Confidence limit. ^4^ Statistically significant values in bold. ^5^ EZPCP = Edmonton Zone Palliative Care Program.

## Data Availability

The data presented in this study are available on request from the corresponding author. The data are not publicly available due to privacy of health information.
